# Aerogels from Cellulose Phosphates of Low Degree of Substitution: A TBAF·H_2_O/DMSO Based Approach

**DOI:** 10.3390/molecules25071695

**Published:** 2020-04-07

**Authors:** Christian B. Schimper, Paul S. Pachschwoell, Hubert Hettegger, Marie-Alexandra Neouze, Jean-Marie Nedelec, Martin Wendland, Thomas Rosenau, Falk Liebner

**Affiliations:** 1 University of Natural Resources and Life Sciences, Vienna (BOKU), Institute for Chemistry of Renewable Resources, Konrad-Lorenz-Straße 24, A-3430 Tulln, Austria; christian.schimper@acticell.at (C.B.S.); paul.pachschwoell@gmail.com (P.S.P.); hubert.hettegger@boku.ac.at (H.H.); thomas.rosenau@boku.ac.at (T.R.); 2 Vienna University of Technology, Institute of Materials Chemistry, Getreidemarkt 9/165, A-1060 Vienna, Austria; marie-alexandra.neouze@anr.fr; 3 Université Clermont Auvergne, CNRS, SIGMA Clermont, ICCF, F-63000 Clermont-Ferrand, France; jean-marie.nedelec@sigma-clermont.fr; 4 University of Natural Resources and Life Sciences, Vienna (BOKU), Institute for Chemical and Energy Engineering, Muthgasse 107, A-1190 Vienna, Austria; martin.wendland@boku.ac.at; 5 Johan Gadolin Process Chemistry Centre, Åbo Akademi University, Porthansgatan 3, FI-20500 Åbo/Turku, Finland; 6 University Aveiro, Department of Chemistry and CICECO Aveiro Institute of Materials, Campus Universitário de Santiago, 3810-193 Aveiro, Portugal

**Keywords:** cellulose phosphate, cellulose phosphate aerogel, interconnected porosity, supercritical carbon dioxide, tetrabutylammonium fluoride, TBAF/DMSO

## Abstract

Biopolymer aerogels of appropriate open-porous morphology, nanotopology, surface chemistry, and mechanical properties can be promising cell scaffolding materials. Here, we report a facile approach towards the preparation of cellulose phosphate aerogels from two types of cellulosic source materials. Since high degrees of phosphorylation would afford water-soluble products inappropriate for cell scaffolding, products of low DS_P_ (ca. 0.2) were prepared by a heterogeneous approach. Aiming at both i) full preservation of chemical integrity of cellulose during dissolution and ii) utilization of specific phase separation mechanisms upon coagulation of cellulose, TBAF·H_2_O/DMSO was employed as a non-derivatizing solvent. Sequential dissolution of cellulose phosphates, casting, coagulation, solvent exchange, and scCO_2_ drying afforded lightweight, nano-porous aerogels. Compared to their non-derivatized counterparts, cellulose phosphate aerogels are less sensitive towards shrinking during solvent exchange. This is presumably due to electrostatic repulsion and translates into faster scCO_2_ drying. The low DS_P_ values have no negative impact on pore size distribution, specific surface (*S*_BET_ ≤ 310 m^2^ g^−1^), porosity (Π 95.5–97 vol.%), or stiffness (*E*ρ ≤ 211 MPa cm^3^ g^−1^). Considering the sterilization capabilities of scCO_2_, existing templating opportunities to afford dual-porous scaffolds and the good hemocompatibility of phosphorylated cellulose, TBAF·H_2_O/DMSO can be regarded a promising solvent system for the manufacture of cell scaffolding materials.

## 1. Introduction

Amplifying efforts towards a more bio-based economy have recently revived the urge for novel smart processes capable of efficiently transforming biomass or its constituents into functional materials. Among the broad variety of biopolymers, cellulose is probably the most valuable renewable resource, since it is an abundant unique source of energy, chemicals, and materials. It is easy to access in high purity [1], biocompatible [2,3], and is not a food competitor.

In context with the increasing awareness for a more responsible use of energy and materials in all areas of life, it is easy to understand that research on lightweight, open-porous, and bio-based materials optimized in stiffness-to-weight proportion has greatly advanced in the last decade [4]. This development has been boosted by the nowadays broader availability of supercritical carbon dioxide technologies. The latter allow—besides chemical modification, coating, or foaming—for largely non-destructive drying of biopolymer gels [5]. This gives access to a new family of ultra-lightweight aerogels which would not be accessible with the same features using classical drying techniques [6,7,8]. Even though not having conquered industrial production yet, cellulose-derived aerogels are promising candidates for a wide range of applications. This includes thermal super-insulation [9], specific sorption of gases or solutes [10], carrier support in catalysis [11], morphological templating [12], energy generation and storage [13], as well as wound dressings [14,15,16], transdermal drug delivery [17,18], or tissue engineering [19,20,21].

Depending on the target application, cellulosic aerogels can be required to feature a broad spectrum of specific properties. The demands are rather simple for thermal superinsulation panels. The latter would require low apparent density, narrow mesopore distribution, sufficient dimensional stability in humid atmosphere, resistance towards microbial degradation, and facile processability [22]. Cell scaffolding materials, however, have to meet complex requirements [19,23,24]. Dual-porosity, i.e., interconnected micron-size pores accommodated in networks of nanoporous struts, and biocompatibility for example are key features. While biocompatibility is inherent to cellulose, dual-porosity can be provided, such as using temporary scaffolds of packed beds of porogens [25]. Besides dual-porous architecture, appropriate nanotopology, surface chemistry, electrical charge density, mechanical properties, or availability of growth factors are further important prerequisites [26]. This is complemented by purity and preservation of chemical integrity of cellulose throughout processing into the desired cell scaffolding materials.

Solution casting and subsequent coagulation of cellulose by an antisolvent is one of the most facile and efficient approaches towards shaped open-porous materials [27,28]. However, the choice of solvents able to solubilize cellulose is rather limited [7]. The complex requirements of cell scaffolding materials in terms of purity, morphology, or surface properties further narrow the range of potentially applicable solvents. Reasons include potential derivatization as demonstrated for common ionic liquids [29], hydrolytic cleavage [30], and formation of undesired by-products [31].

Recently we reported about the impact of different cellulose solvents and antisolvents on nanomorphological and -topological features (e.g., crystallinity, size and shape of pores, dimension, organization, and surface roughness of network building nanoparticles) of cellulose II aerogels [32]. Both nanomorphology and nanotopography play a crucial role in tissue engineering. This has been recently demonstrated for neurite extension by neuronal PC12 cells grown on collagen-coated mesoporous silica aerogels [33,34] and on electrically conductive carbon aerogels [35]. Based on the finding that the non-derivatizing solvent system tetrabutylammonium fluoride / dimethylsulfoxide (TBAF/DMSO) affords the formation of particularly small particles, and hence, finely substructured cellulose II networks [32], we extended the exploration of this solvent for processing different types of pulp into cellulose aerogels [36]. Simultaneously we were aiming to improve the dissolution performance of TBAF·xH_2_O/DMSO. Optimization of the water-to-fluoride ratio was one target, since the latter is a sensitive parameter governing dissolution kinetics. It turned out that the optimum of cellulose dissolution is reached at water-to-fluoride molar ratios of (0.8 ≤ χ_wf_ ≤ 2). Water contents outside this range impede cellulose dissolution either by *E2*-type Hofmann decomposition of TBAF into but-1-ene, tributylamine and thermodynamically stable HF_2_^-^ ions (χ_wf_ ≤ 0.8) [37] or simply by insufficient rearrangement capabilities for the hydrogen bonding network in cellulose (χ_wf_ ≥ 2) [38].

This study investigates the utilization of TBAF·xH_2_O/DMSO for the preparation of cellulose phosphate aerogels since the latter have shown promise as cell scaffolding materials. Recently, cross-linked aerogels obtained from cellulose nanocrystals carrying a low count of phosphate halfester groups on their surface were reported to fulfil many requirements of viable bone tissue scaffolds [21]. This complements the results of earlier attempts aiming at the preparation of cellulose II phosphate aerogels via shaping and coagulation of cellulose phosphates from *Lyocell* dopes, i.e., using the solvent system *N*-methylmorpholine-*N*-oxide (NMMO)/water. Already at low degrees of phosphorylation (DS_P_ ca. 0.2), biomineralization and good hemocompatibility in terms of hemostasis and inflammatory response were observed [39]. However, some shortcomings of NMMO related to its elevated melting point, its proneness towards autocatalytic degradation (in the presence of acidic phosphate groups) and the specific solidification behavior of cooling dopes were reasons enough to look for alternatives.

This study investigates the preparation of cellulose phosphate aerogels using TBAF·xH_2_O/DMSO as a non-derivatizing solvent for the preparation of cellulose phosphate aerogels of low degree of phosphorylation. It has been tested with the example of two representative cellulosic source materials. The exploratory work was expected to provide data for developing a cellulose phosphate 3D printing approach for cell scaffolding materials.

## 2. Results and Discussion

### 2.1. Phosphorylation

Phosphorylation of the two selected cellulosic source materials of this study—cotton linters (CL) and eucalyptus prehydrolysis kraft pulp (hwPHK)—was accomplished using concentrated phosphoric acid. Triethyl phosphate was used as solvent and phosphorus pentoxide served as a reactive binder for water released during esterification. Microwave-assisted pressure digestion in HNO_3_/H_2_O_2_ and subsequent ICP-OES analyses confirmed that the desired low degrees of phosphorylation were obtained. While the degree of substitution by phosphate moieties (DS_P_) was 0.18 for the cotton linters sample (corresponding to a phosphorous content of 33.4 g kg^−1^; Table 1), it was 0.24 for hwPHK (49.1 g kg^−1^). At this low degree of phosphorylation, the cellulose derivatives were proven not to be water-soluble yet. This is a prerequisite for cellulose coagulation from solution state, such as in NMMO/H_2_O or TBAF/DMSO, triggered by the addition of the anti-solvent water. Dissolution in aqueous media is also not desired for any use as cell scaffolding material. ^31^P NMR as exemplarily conducted for the hwPHK sample confirmed the expected selective substitution of the primary alcohol groups in C6 position of the anhydroglucose units (data not shown).

Previously, we have shown that a wide range of cellulosic source materials can be dissolved up to 3 wt.% cellulose content within comparatively short dissolution time [36]. Optically clear solutions of sufficiently low viscosity, and hence, good workability, were obtained. Targeting solution casting, a solid content of 3 wt.% was envisaged for the cellulose phosphates of this study, too.

**Figure 1:**
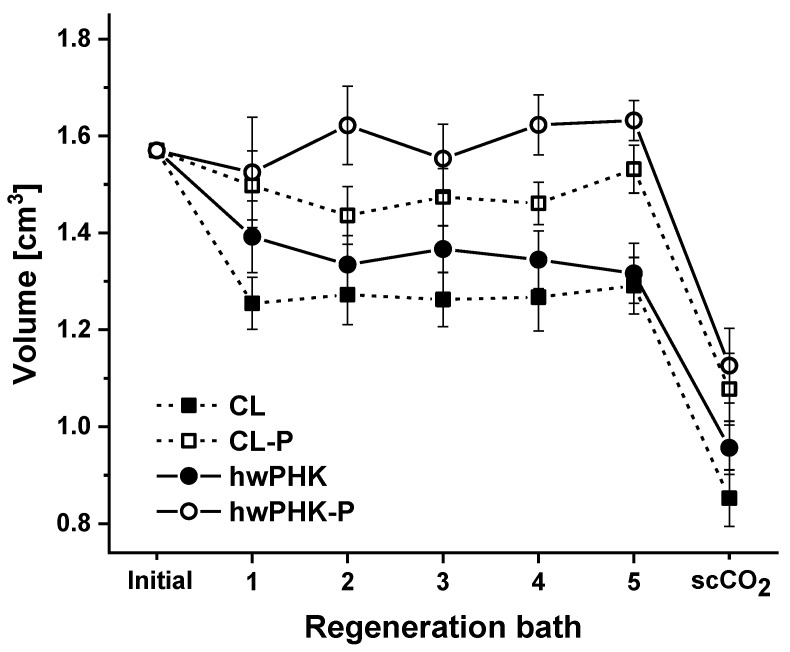
Change in volume of the gels during regeneration in ethanol followed by supercritical carbon dioxide (scCO_2_) drying (-P indicates phosphorylated samples). Error bars indicate the 95% confidence interval.

Like their non-derivatized counterparts, the two types of cellulose phosphates could be easily dissolved at the target concentration of 3 wt.% in DMSO that contained 16.6 wt.% TBAF and 0.95 wt.% H_2_O. Visually and microscopically clear solutions were obtained within 4 h of dissolution at 60 °C. Solution casting of the comparatively low-viscous solutions and subsequent submersion of the molds in water afforded self-standing transparent hydrogels. According to common practice, the hydrogels were subjected to a series of solvent exchange steps aiming to incrementally increase the ethanol content up to 100% to prepare the gels for supercritical carbon dioxide (scCO_2_) drying. Since dimensional stability is a key property for many applications, and particularly challenging to achieve for ultra-lightweight biopolymer-derived polysaccharide materials, possible swelling and shrinking throughout all steps of the aerogel preparation procedure were assessed. It was shown that coagulation and hydrogel formation, respectively, occurred across the entire cast solution as evident from the shape and dimensions of the free-standing hydrogels obtained. However, slight shrinkage by syneresis was observed when the gels were left in the PTFE molds, such as for 72 h. Interestingly, this was not the case when cardboard molds were used [40]. Immersion of the CL and hwPHK hydrogel samples in water of incrementally increasing ethanol content (50%, 75%, 96%, and 100%) caused the gels to shrink by up to 18 vol.% (Figure 1, Table 1). This is in agreement with previous work [36] and has been observed for other cellulose II gels too. Examples include gels formed by water-induced coagulation of cellulose from solvent systems like NMMO·H_2_O, [EMIm][OAc]/DMSO, or Ca(SCN)_2_⋅8H_2_O/LiCl [32].

Interestingly, both types of hydrogels prepared from the respective phosphorylated cellulosic materials suffered from less shrinkage during the solvent exchange sequence than their P-free counterparts (Figure 1). This is presumably due to the presence of negatively charged phosphate groups on the surface of cellulose II nanoparticles that form and aggregate spontaneously during coagulation to afford three-dimensional networks [7]. Repulsive forces and possibly steric hindrance can partially impede supramolecular arrangement of cellulose chains. This could explain why virtually no shrinkage—in case of hwPHK-P even minimal swelling—was observed after completing the solvent exchange (Table 1). Significant shrinkage of all gels, however, was caused by the final scCO_2_ drying step. In sum of the entire solvent exchange and scCO_2_ drying process, the volume loss for the resulting aerogels was about 39–46% for the non-derivatized cellulosic materials and 28–31% for their phosphorylated counterparts. A similar extent of shrinkage (29 vol.%)—occurring mostly during the final scCO_2_ drying step—has been recently reported for anisotropic cellulose II gels. The latter were obtained by self-assembly of cellulose (3 wt.%) in super-cooled 1,1,3,3-tetramethylguanidinium acetate under the impact of decelerated antisolvent infusion [28]. As discussed earlier [12], shrinkage is governed by solvent-polymer as well as polymer-polymer interactions. They can be quantified by cohesive energy *E*_coh_ or cohesive energy density *e*_coh_ when *E*_coh_ is related to unit volume. The square root of *e*_coh_ and Hildebrand solubility parameter δ_H_, respectively, is frequently used to predict solvent–polymer interactions. Since *E*_coh_ represents the sum of contributions by dispersion forces, permanent dipol-dipol forces, and hydrogen bonding, any variation of polymer-solvent composition potentially impacts these interactions. While the changes in composition throughout the lengthy solvent exchange procedure are rather small, scCO_2_ drying causes rapid changes in composition of the interstitial fluids. This is most pronounced in the initial stage of drying. Molecular dynamics simulations have recently shown that the solubility parameters change significantly with enrichment of scCO_2_ by co-solvents, such as ethanol. It changes in a reverse way when the volume fraction of co-solvent is reduced [41]. For pure scCO_2_ (and pure co-solvent), δ_H_ decreases with temperature and rises with pressure and density. Addition of co-solvents, such as ethanol, increases δ_H_ significantly, boosting with the amount of added co-solvent. Considering the different stages of scCO_2_ drying, i.e., i) CO_2_ pressurization, ii) transition from liquid to supercritical state causing considerable changes in density, iii) formation and elution of a scCO_2_-expanded ethanol phase of rapidly decreasing ethanol content, and iv) final depressurization, it is evident that this part of the aerogel preparation is the most sensitive part with regard to shrinkage, specifically for ultra-lightweight aerogels. Beyond that it is assumed that small quantities of structural water—which acts as a softener for the cellulose II networks—are released towards the end of the drying procedure. This is caused by condensation of labile hydroxyl-groups which seems to start already when the gels are transferred to absolute ethanol since most of the gels stiffened slightly during this stage, comparable to hornification of cellulose at elevated temperature.

According to the different extent of shrinkage throughout the solvent exchange and scCO_2_ drying procedure, somewhat lower bulk densities were obtained for the cellulose phosphate aerogels. While the values of the latter were largely similar (47 vs. 50 mg cm^−3^), that of their counterparts from non-derivatized cellulose varied to a larger extent and ranged from 58 (CL) to 71 mg cm^−3^ (hwPHK, Table 1).

### 2.2. Chemical Integrity of Cellulose Phosphates during Dissolution

In an attempt to verify whether the chemical integrity of the cellulose phosphates was preserved throughout dissolution, both CL-P and hwPHK-P were subjected to elemental analysis prior to and after dissolution in TBAF⋅H_2_O/DMSO (20 °C, 16 h). The results revealed a significant loss of phosphate groups for both of the samples. While for CL-P, the DS_P_ value decreased from 0.18 to 0.13 (−28%) and was more pronounced for hwPHK-P (−55%; DS_P_ 0.209 vs. 0.095). Kinetic studies, as exemplarily conducted for the hwPHK-P sample, showed that the losses occur mainly in the initial stage of dissolution since the DS_P_ values remained largely constant after two hours of dissolution time (Table 2).

This suggests that for hwPHK about 55% of the presumably introduced phosphates were not covalently bonded but resisted being trapped in the phosphorylated cellulose. Even if released during dissolution in TBAF/DMSO, it was trapped again upon water-induced coagulation, but was then largely removed during the various solvent exchange steps. This conclusion is supported by the fact that identical final DS_P_ values (0.096) were obtained after 8 h of dissolution time at both room temperature and 60 °C. Aiming to exclude interference of TBAF, the hwPHK-P dissolution experiment was repeated, however, using DMSO as solvent only (4 h, room temperature). Following repeated washings with ethanol and vacuum drying, a remaining P content of 71% was found. Altogether, this implies that the used washing procedure after phosphorylation (*n*-hexanol, ethanol, water) as proposed elsewhere [42] is not efficient enough. The results were also a motivation to re-check similar hwPHK-P retention samples of a previous study for any possible reduction of DS_P_. These samples had been used for preparation of potential cell scaffolding materials via the *Lyocell* route [39]. Cellulose was here dissolved in molten *N*-methylmorpholine-*N*-oxide monohydrate (NMMO·H_2_O) at 100 °C using propyl gallate and *N*-benzylmorpholine-*N*-oxide (NBnMO) as stabilizers. Repeating dissolution of the hwPHK-P retention samples (DS_P_ = 0.25) in NMMO·H_2_O at 110 °C, subsequent coagulation by ethanol and final scCO_2_ drying revealed that also in this dissolution/coagulation approach the DS_P_ decreased; however to a lesser extent (33%, DS_P_ = 0.17). A DS_P_ value of 0.22 was obtained (13% loss) when the hwPHK-P sample was extracted with ethanol only at room temperature.

### 2.3. TBAF Content of Cellulose Phosphate Aerogels

**Figure 2:**
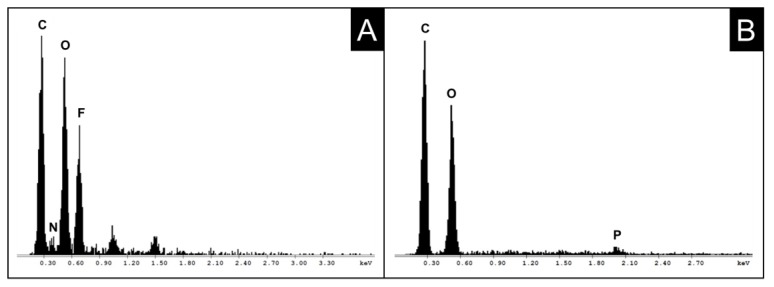
EDAX spectra of aerogels obtained without (**A**, non-derivatized CL) and after (**B**, phosphorylated CL) implementing a H_2_O washing step prior solvent exchange and scCO_2_ drying.

Even though TBAF/DMSO is considered a direct, non-derivatizing cellulose solvent, selected aerogel samples were subjected to nitrogen and fluorine analysis by energy-dispersive X-ray spectroscopy (EDAX). While for CL and hwPHK aerogels low nitrogen (and fluorine) contents close to the detection limit were determined (0.05% N, data not shown), the latter were significantly higher for their phosphorylated counterparts (Figure 2A). Independent of the cellulosic source material and degree of phosphorylation (CL-P DS_P_ = 0.18; hwPHK-P DS_P_ = 0.24), respectively, significantly higher values of 0.74% N were obtained. Considering both the nominal DS_P_ values of the two phosphorylated cellulosic source materials (DS_P_ = 0.2 on rough estimate) and the real DS_P_ ones (DS_P_ = 0.09 = 45% of the nominal DS_P_), it can be concluded that about all covalently introduced phosphate moieties carry one ammonium counter ion. The over-proportional residual content of fluoride ions is difficult to explain. Likely reasons could be remnants of phosphoric acid competing with fluorine for TBA cations or partial degradation of TBAF (triggering release of tributylamine, but-1-ene and the thermodynamically stable HF_2_^−^ ions; cf. above) and preferred removal of the nitrogenous compounds during solvent exchange and scCO_2_ drying.

Covalent immobilization of both nitrogen and fluorine, however, can be ruled out since repeated washing of the samples with deionized water (3×) afforded EDAX spectra free of any N and F signals (Figure 2B).

### 2.4. Aerogel Morphology

**Figure 3:**
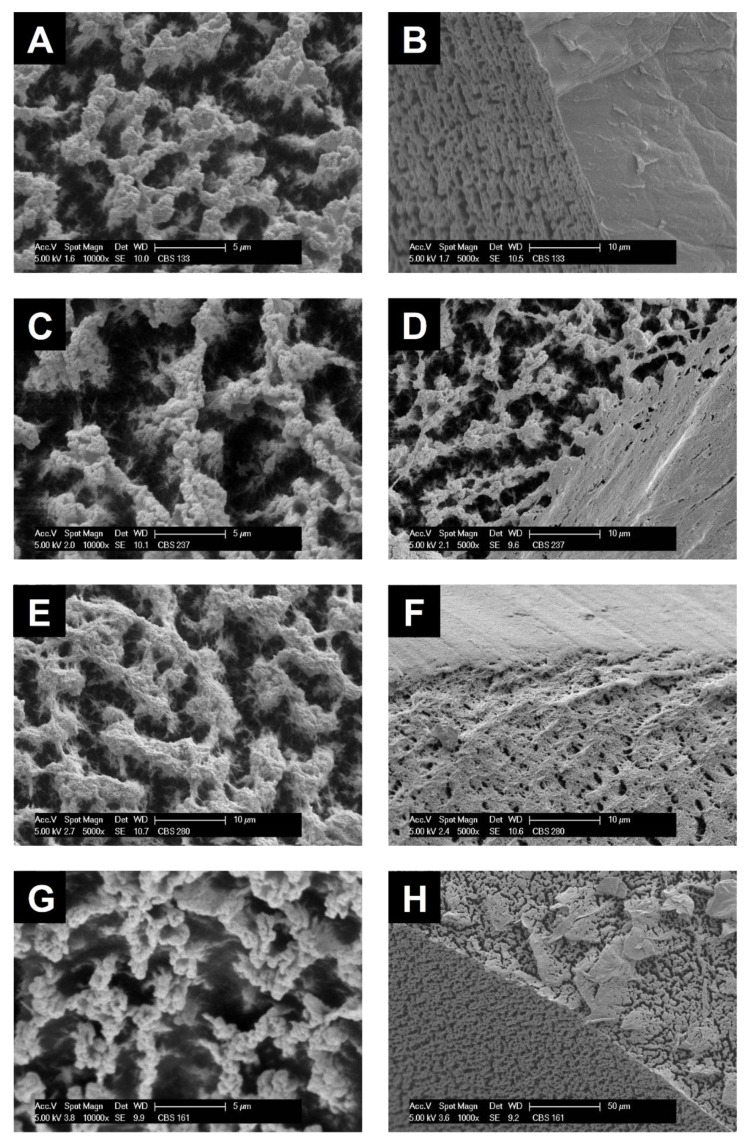
SEM micrographs of the interior (**A**, **C**, **E**, **G**) and near-surface breaking edge (**B**, **D**, **F**, **H**) of aerogels from non-derivatized cellulose (CL: **A**, **B**; hwPHK: **E**, **F**) and their phosphorylated counterparts (CL-P: **C**, **D**; hwPHK-P: **G**, **H**) at different magnification.

Selected aerogel samples were split open by gently pulling apart the two halves of the cylindrical specimen using a small fork with narrow teeth. Scanning electron microscopy (SEM) of the interior of these aerogels revealed the presence of an isotropic network largely composed of interconnected spherical particles (Figure 3). Its appearance resembles that of other cellulose gels formed by spinodal decomposition, such as from cellulose solutions in ionic liquids (e.g., [EMIm][OAc]) or molten NMMO·H_2_O) [43,44]. The isotropic networks are formed from (partially elongated) single-digit micron-sized clusters of cellulosic spheres being a few hundred nanometers in diameter. The voids in between the clusters are interconnected and of similar spatial shape and dimension (diameter 2–4 µm, partially elongated). A closer look revealed the coexistence of a further substructure for all of the studied aerogels. In particular, the micrograph of Figure 3E suggests that the spherical submicron particles are composed of finer fibrils, which is in agreement with previous studies [32,36]. Phosphorylation at the low degrees of substitution envisaged in this study had, however, virtually no impact on microscale morphology (*cf.* Figure 3A vs. Figure 3C and Figure 3E vs. Figure 3G).

SEM micrographs of the cutting edges close to the exterior surface revealed that a comparably dense skin had formed in case of the aerogels from the non-derivatized cellulosic materials. It has an average thickness of 10–50 µm and is deficient in micron-size pores (Figure 3B and Figure 3F). Skin formation is evidently less pronounced in the cellulose phosphate aerogels. In particular, the hwPHK-P sample features a largely skin-free open-porous flaky structure (Figure 3H). Skin formation is a well-known phenomenon occurring during cellulose processing from solution state, such as in the course of wet spinning of *Lyocell* dopes. Different coagulation kinetics across the diameter of the extruded dope strands result in significant morphological variation, typically comprising of a compact fiber core, a porous middle zone, and a semipermeable fiber skin [45].

Although relying on different coagulation mechanisms, also membranes prepared from cellulose solutions in mixtures of sodium hydroxide and urea exhibited morphological differences between surface and core of the respective materials. Their extent differed with the type of antisolvent used for cellulose coagulation and was most pronounced for ethanol [46]. Formation of a layered structure comprising of denser outer and looser inner zones has been shown also for cellophane films (sheet-extruded viscose rayon; [47]). Anisotropic cellulose II aerogels (Hermans orientation factor 0.46) obtained by self-assembly of cellulose in super-cooled ionic liquid under the impact of decelerated antisolvent infusion [28] feature skin formation too.

### 2.5. Compression Testing

Uniaxial compression testing and evaluation of the respective stress–strain relationships confirmed the typical compression behavior of lightweight cellulose II aerogels as discussed elsewhere [32]. Besides ductility, low rigidity as expressed by Young’s modulus (*E* ≤ 14.5 MPa), and absence of sample buckling (zero Poisson ratio), the stress-strain curves exhibit a pronounced plateau region (10–50% strain). In this region, compression energy is dissipated by gradual collapsing of the cellular structure. Beyond that plateau, strain hardening sets in. Recently, it has been shown for aerogels from nanofibrillated 2,3-dicarboxyl cellulose that strain hardening can coincide with pore size harmonization in favor of mesopores giving access towards superinsulating aerogels [9]. Interestingly, both Young’s modulus and density-normalized specific Young’s modulus of the CL aerogel were higher than that of comparable CL aerogels obtained using NMMO·H_2_O or [EMIm][OAc]/DMSO as cellulose solvent (Table 3; [32]). Phosphorylation, at least at the envisaged low degrees of substitution, had no clear impact on the anyway low values of Young’s modulus *E*, yield stress (σ_y_) and yield strain (ε_y_). While *E*_ρ_ and ε_y_ slightly increased with phosphorylation for the aerogels derived from cotton linters, it was the reverse for the hwPHK samples.

Based on the apparent density (ρ_A_) of the aerogels and assuming a skeletal cellulose density of ρ_S_ = 1.56 g cm^−3^, porosity (Π) values ranging between 95.46 and 96.99% were calculated using the following equation: Π (%) = 1 – ρ_A_ / ρ_S_ (Table 4). At a first glance, these values combined with the information of the SEM pictures (Figure 3) could be perceived as indicative for the presence of very low specific surfaces only in all prepared aerogels. However, nitrogen sorption experiments at 77 K provided a different picture. Evaluation of the isotherms in the low relative pressure range (p/p^0^ = 0.05–0.2) of the adsorption branches using the Brunauer–Emmett–Teller (BET) approach revealed a considerable monolayer nitrogen adsorption. It corresponds to about 350–370 m^2^ g^−1^ which is almost as high as reported for anisotropic mesoporous aerogels of narrow size distribution [9]. These relatively high values are indicative of the presence of a well-developed nanoporous substructure not visible in the micrographs of Figure 3. Somewhat lower specific surface values were calculated for both types of phosphorylated cellulose aerogels (Table 4). However, these results require careful treatment since gas sorption in aerogels of multiscale porosity depends on many factors. It also includes the C factor of the BET equation. Its significance is low since the C factor—in the strict sense—is a measure of interaction between a non-porous surface and adsorbent molecules [48]. Assuming largely similar morphology, however, the significantly lower C values obtained for the phosphorylated samples can be interpreted as a considerable drop in interaction due to the introduction of the polar phosphate moieties.

All sorption isotherms confirmed the presence of a considerable volume fraction of mesopores as strongly evident from their IUPAC type IV shape [49]. The latter is characterized by slow monolayer adsorption at relatively low relative pressure (p/p^0^ ≤ 60 kPa), but higher ad- and desorption rates beyond that (0.07 ≤ p/p^0^ ≤ 0.1 MPa).

**Figure 4:**
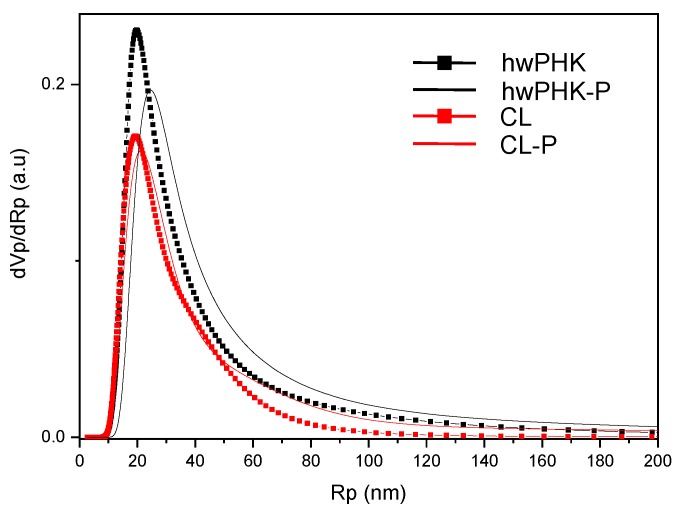
Normalized pore-size distributions as obtained by thermoporosimetry.

Due to the fragility of lightweight cellulosic aerogels, pore size distributions representing the true porosity are methodologically difficult to obtain, in particular for aerogels of multiscale pore size distribution. While mercury intrusion is not applicable (the high specific density causes pore collapsing), other methods are limited to a certain range of pore size. Evaluation of data points taken from the desorption branches of the nitrogen sorption isotherms using the Barrett–Joyner–Halenda model (BJH) suggested the presence of mesoporous domains. The latter are characterized by a narrow pore size distribution peaking at around 9–11 nm. They seem to exist in all studied materials. The results of the BJH calculations, however, also indicate the presence of larger pores. This is in agreement with the transition of the two branches of the adsorption isotherms at p/p^0^ = 0.1 MPa and the SEM micrographs. Thermoporosimetry studies confirmed both the presence of mesopores and coexistence of significantly larger pores (Figure 4). All pore-size distributions have their maximum at about 50 nm diameter. The results of the nitrogen sorption experiments, thermoporosimetry data, and SEM micrographs suggest that phosphorylation at the studied low DS_P_ values has only a marginal impact on morphological features. This includes specific surface and pore-size distribution.

### 2.6. Aerogel Stability and Moisture Sorption during Storage

**Figure 5:**
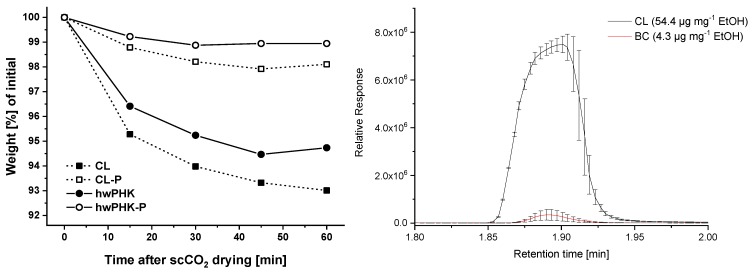
Weight [%] of the materials recorded over 60 min after scCO_2_ drying (left). Release of ethanol from freshly scCO_2_ dried cotton linters aerogels (CL) and bacterial cellulose aerogels (BC) under headspace (80 °C) GC-MS conditions (right).

The abundance of hydroxyl groups renders cellulosic surfaces very sensitive towards moisture sorption. While this feature is highly desired in some applications like wound dressings [15], it is the opposite for lightweight cellulose aerogels. Their high volume fractions of interconnected nanopores exceeding sometimes 99 vol.% render them very fragile, in particular in moist environments. Capillary condensation is a phenomenon that occurs in hydrophobic materials as well, but is amplified by huge internal surfaces of high hydrophilicity. Water condensation in capillaries give rise to the formation of strong inward forces alongside a capillary gradient adjacent to the water menisci formed. These forces are caused by differences in specific energies of the involved phases. Their strength is reversely correlated with pore radii, which renders open-nanoporous, hydrophilic and soft materials particularly sensitive towards shrinkage. Considering both the anhydrous conditions in the last stages of solvent exchange and scCO_2_ drying, as well as the hydrophilicity of the studied cellulosic materials, it was assumed that the aerogels would gain weight immediately once exposed to air. However, this was not the case. It turned out that the aerogels prepared from the non-derivatized source materials lost about 5–7 wt.% instead within 60 min after scCO_2_ drying (Figure 5). This is surprising at a first glance and needed a more thorough investigation since this weight loss could be explained solely by degassing of CO_2_ and replacement by air. Considering a bulk density of 71.2 mg cm^−3^ for the CL aerogels for example and a cellulose skeletal density of 1.56 g cm^−3^, the corresponding pore volume would be about 0.95 cm^−3^. Assuming ideal gas behavior, standard conditions and occupation of the entire pore volume by CO_2_, its quantitative replacement by dry air (80% N_2_ and 20% O_2_) would cause a weight loss of ca. 1.22 mg. This would be equivalent to 1.7% loss related to the bulk density calculated from weight and spatial dimensions shortly after scCO_2_ drying. Even if water sorption would have caused the difference between measured bulk density (71.2 mg cm^−3^) and theoretical density (55.25 mg cm^−3^)—calculated from the original cellulose content of the CL gel (3 wt.%) and the volume loss throughout aerogel preparation (45.7 vol.%)—the maximum possible weight loss would be 2.21 wt.% only. Therefore, it is reasonable to conclude that small quantities of ethanol were still present in the scCO_2_ dried aerogels, which is in agreement with olfactory impressions. Headspace-GC/MS investigations confirmed this assumption. They revealed that—depending on the cellulosic material processed into aerogels—considerable amounts of ethanol can remain after scCO_2_ drying. While scCO_2_ extracted virtually all ethanol from the pores of bacterial cellulose aerogels of similar sample size and shape within one hour drying time, more than the ten-fold amount of ethanol remained for the significantly denser CL aerogels. Phosphorylation, even at low degree of substitution, seems to disfavor ethanol sorption as evident from the slight weight losses after scCO_2_ drying.

Long-term dimensional stability and moisture sorption of all aerogels were studied for a time period of 84 days and controlled levels of relative humidity. Weight gain and shrinkage values revealed that moisture uptake occurs for all studied aerogels mainly during the first 24 h (data not shown) and is largely completed after 48 h. This was independent of cellulose type, degree of phosphorylation, and relative humidity (Table 5) except for the highest value of 98%RH. Here, water sorption increased by 2.2 wt.% for the CL sample from 23.6% after 2 days to 25.8% after 84 days. The amount of water adsorbed was confirmed to depend on the air moisture content. While at 30% RH, water uptake was negligible for all samples, it was moderate at 65% RH (5.0–6.6%) but strong at 98% RH (23.6–24.2%). Corresponding to the amounts of adsorbed water, all samples suffered from significant shrinkage, specifically within the first two days of storage. Here, the extent of shrinkage increased strongly in the order 30%RH (24–28%) < 65%RH (64–73%) < 98%RH (90–93%). Storage in anhydrous environment as accomplished by placing the samples in a closed compartment over P_4_O_10_ is not recommended. Removal of structural water and hornification cause the aerogels to shrink at an extent comparable to that observed for 30% RH.

As a result of water uptake and shrinkage, the bulk densities of the prepared aerogels increased as well. While it was to a minor extent only when storing them at 0% and 30% RH, three- (65% RH) to 17-fold (98% RH) higher values were obtained for the more humid environments. Phosphorylation rendered the aerogels somewhat more sensitive towards moisture sorption and shrinkage.

### 2.7. Thermostability

**Figure 6:**
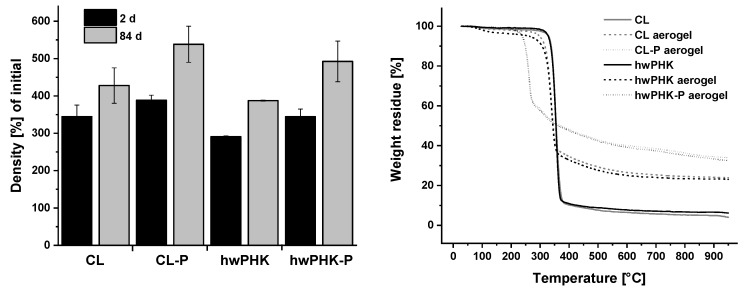
Bulk density [%] of phosphorylated and non-derivatized aerogels after two and 84 days during conditioning at 65% RH (left). Thermogravimetric analysis of cellulose II aerogels from non-derivatized and slightly phosphorylated cotton linters (CL, CL-P) and hardwood prehydrolysis kraft pulp (hwPHK, hwPHK-P). TGA profiles of the unprocessed source materials were recorded for reference purposes (right).

Thermogravimetric analysis (TGA) conducted in helium atmosphere revealed some interesting differences between the different sets of samples. This applies not only to the cellulosic source materials and the aerogels obtained thereof. It refers also to the comparison between aerogels prepared from non-derivatized cellulose and their phosphorylated analogues (*cf.* Figure 6, right). The two non-processed CL and hwPHK samples were virtually fully stable up to about 325 °C. Here, prominent polymer degradation sets in, exactly as reported for cotton linters before [50]. Thermal degradation is largely completed at 350 °C already when about 90% of the initial weight has been released as volatiles. Continued pyrolysis and carbonization up to 950 °C caused a further 50% reduction of the remaining mass.

Processing of the cellulosic source materials into lightweight open-porous aerogels gives rise to a series of differences in material properties. Compared to the cellulosic fibers of the source materials, the diameters of the aerogel network forming fibrils is much smaller [36] and the degree of crystallinity lower [32]. This translated into much higher specific surface areas and moisture sensitivity. Nanostructuration furthermore imparts thermal insulation properties to aerogels presumably considerably retarding heat-transfer. These properties are assumed to increase the sensitivity of respective aerogels towards temperature alterations and may promote kinetically less favored side reactions. This is supported by the following observations: (i) the above-described humidity-dependent adsorption of water is well reflected by smaller weight losses (ca. 2–4 wt.%) in the temperature range ≤ 150 °C; (ii) intra- and intermolecular condensation occurring between 150 and 240 °C [51] give rise to further weight loss of 1–2 wt.%; (iii) depolymerization starts at 300 °C already likely due to reduced crystallinity, and (iv) the mass remaining at 350 °C (36–37 wt.%) and 950 °C (24–25 wt.%) was much higher compared to the non-processed cellulosic source materials. The latter might be a result of stabilization by cross-linking and hornification.

The TGA profiles of the CL-P and hwPHK-P aerogels differed strongly from that of their non-derivatized analogues despite the low degrees of phosphorylation. While desorption of physically bonded water was negligible for the CL-P and hwPHK-P samples, prominent weight reduction started at 235 °C already. However, only about 36% of the initial weight was lost in this step, which is much lower compared to the cellulosic source materials (90 wt.%). Formation of volatiles continued at significantly lower rates until the final temperature of 950 °C and a residual weight of about 40% relative to the initial weight of the samples was reached. It is assumed that acidic phosphate groups as present in CL-P and hwPHK-P are capable to boost formation of low-molecular compounds. This includes levoglucosan, which has been shown to be involved in re-polymerization of volatile cellulose pyrolysis products and secondary char formation (*cf.* [52,53]). The latter could give rise to the formation of thin, heat-shielding char layers efficiently preserving a reasonably large weight fraction under enhanced pyrolytic conditions.

## 3. Experimental Section

Phosphoric acid (99%), phosphorus pentoxide (98.5%), triethyl phosphate (99.8%, all Sigma-Aldrich, Schnelldorf, Germany), *n*-hexanol (98%, Acros Organics, Geel, Belgium), tetra-*n*-butylammonium fluoride (TBAF) trihydrate (≥97%, Sigma-Aldrich, Schnelldorf, Germany), dimethyl sulfoxide (DMSO, ≥99.9%, Merck, Darmstadt, Germany), molecular sieves 4 Å (Roth, Karlsruhe, Germany), and ethanol (96 vol.% and absolute, Merck, Darmstadt, Germany) were of highest available grade.

Cotton linters (CL) and total chlorine-free bleached hardwood (eucalyptus) pre-hydrolysis kraft pulp (hwPHK) were provided by collaboration partners of the COST E41 action. Their weight average molecular weight (M_W_) as well contents of carbonyl group were determined as detailed elsewhere [36,54]:

- CL: M_W_ 143.2 kg mol^−1^, 4.4 mmol g^−1^ C=O (fluorescence labelling of cellulose using carbazole-9-carbonyl-oxy-amine, CCOA;
- hwPHK: M_W_ 154.8 kg mol^−1^, 8.1 mmol g^−1^ C=O;

Prior to dissolution or phosphorylation, the cellulosic source materials were activated by disintegration in water (solid-to-liquid ratio 1:400, wt./wt.). The obtained slurry was freeze-dried and stored at +4 °C until further processing.

### 3.1. Phosphorylation of Cellulose

Phosphorylation was accomplished as described elsewhere [39,42,55]. In brief, 250 g of phosphorus pentoxide was placed under argon atmosphere protection in a 1 L 3-necked round-bottom flask equipped with air-tight mechanical stirrer, condenser, and dropping funnel. Phosphoric acid (354 g) was added portion-wise under external ice-cooling. Triethyl phosphate (185 mL) was added slowly within 6 h. A clear, highly viscous solution was obtained after about 48 h of continued stirring. The thus prepared phosphorylation reagent (PR) was stored in anhydrous atmosphere at +4 °C until further use. A defined amount of cellulosic source material (typically 1.0 g) was activated for phosphorylation by repeated short-time (10 s) disintegration in a large excess of water (1:400, *w/v*). This was followed by submersion in consecutive bathes of ethanol and *n*-hexanol (ca. 1:80, *w/v*; 2 times á 24 h for each of the organic solvents). About 40 mL of the supernatant *n*-hexanol was then decanted by gentle squeezing and replaced by an equal volume of PR. The flasks containing the reaction mixtures were then mounted onto a heated horizontal shaking device and left at 50 °C for 72 h. After that, phosphorylated cellulose was separated from the liquid phase and consecutively washed twice with *n*-hexanol, ethanol, and eventually deionized water. The last step was repeated until the filtrates showed a negative molybdenum blue reaction. The degree of phosphorylation (DS_P_) was analyzed as suggested previously [56]. An aliquot of the sample (100 mg) was subjected to microwave-assisted pressure digestion in HNO_3_/H_2_O_2_ using the following temperature program: 25 → 85 °C (3 °C min^−1^), 85 → 145 °C (ca. 1.5 °C min^−1^), 145 → 200 °C (ca. 1 °C min^−1^, 12 min hold), cooling to room temperature. The phosphorous content of the obtained digestion liquor was analyzed by ICP-OES and used to calculate the DS_P_ according to Equation (1) [56].

(1) $$DS_{p}=\frac{162x}{3100-84x}$$

Finally, an aliquot of phosphorylated cellulose was subjected to solid-state ^31^P and ^13^C NMR experiments to confirm covalent introduction of monophosphate groups.

### 3.2. Preparation of the Cellulose Solvent System TBAF (16.6 wt.%) / H_2_O (0.95 wt.%) / DMSO

TBAF trihydrate (157.55 g) was dissolved in anhydrous DMSO (342.45 g). Then, 325 g of freshly dried molecular sieves 4 Å (600 °C, 3 h, argon atmosphere) was added portion-wise over a period of 96 h to bind about 50% of the crystal water. When the remaining water content as determined by Karl Fischer titration reached about 1.5 wt.%—coinciding with the onset of a faint yellowish (λ = 420 nm) coloration [36]—the drying agent was filtered off. The solution was stored at +4 °C until further use. Immediately before cellulose dissolution, the above-prepared TBAF/DMSO solution was diluted with anhydrous DMSO as described previously to obtain a final TBAF content of 16.6 wt.%. The final water content was 0.95 wt.% which ensures good dissolution performance and largely avoids decomposition of TBAF [36].

### 3.3. Cellulose Dissolution, Casting, Coagulation, Solvent Exchange, and scCO_2_ Drying

The respective non-derivatized and/or phosphorylated cellulosic materials (3 g) were dissolved portion-wise in the pre-heated (60 °C) and continuously stirred solvent system (97 g) to give a 3 wt.% solution. While the obtained cellulose solutions were visually clear after two hours already, optical microscopy (magnification 100×) revealed that full dissolution requires 3–4 h. After four hours, the obtained dopes were cast into molds of cylindrical geometry (Ø = 10 mm, l = 20 mm). The molds were then immersed in 96 vol.% ethanol (EtOH) to initiate cellulose coagulation using a dope-to-ethanol volume ratio of 1:50. After 24 h of residence time, the molds were opened to expose the cylindrical, free-standing lyogels. The latter were transferred into consecutive bathes of 96 vol.% ethanol (3 × 24 h) and absolute ethanol (2 × 24 h) ensuring a gel-to-liquid ratio of 1:20 (*v*/*v*). Drying of the transparent gels was performed using supercritical carbon dioxide equipment (scCO_2_). Samples were loaded in a 500 mL autoclave equipped with two separators for carbon dioxide recycling (Separex SF1, Separex, France). After heating and pressurization to 40 °C and 10.5 MPa, the samples were dried at constant CO_2_ flow (2.5 kg h^−1^) for one hour following recommendations of previous studies [57,58]. It has been shown that increasing the pressure beyond 10 MPa slightly reduces the remaining volume [30]. This is similar for increasing the temperature, as hornification and loss of tightly bonded surface water reduce interfibril distances.

Once ethanol was quantitatively extracted from the voids, the system was slowly and isothermally depressurized to prevent pore collapsing and condensation by Joule–Thomson cooling [59]. Both volume (±0.1 mm^3^) and weight (±1 mg) of the samples (5 replicates) were recorded after each step of the sequential solvent exchange and after scCO_2_ drying.

## 4. Analyses

Scanning electron micrographs of both external and internal aerogel surfaces were taken in different magnification after gold sputtering. A Philips ESEM XL30 or a Quanta 250 FEG with energy dispersive analysis X-ray (EDAX) mapping was used, both operated at an acceleration voltage of 5 kV. Evaluation of the surfaces with regard to remaining traces of TBAF was accomplished by detection of nitrogen and fluorine in EDAX mode.

Nitrogen sorption experiments at 77 K were performed using a Micrometrics ASAP 2020 analyzer. All samples were evacuated overnight at room temperature prior to the measurements. Specific surface areas were calculated according to the Brunauer–Emmett–Teller equation using a set of data points of the respective adsorption branches of the isotherms [60]. Average pore diameters were calculated from data points of the desorption branches using the Barrett–Joyner–Halenda (BJH) approach [61]. Nitrogen sorption experiments were complemented by thermoporosimetry using a Mettler-Toledo DSC 823e differential scanning calorimeter (DSC) equipped with liquid nitrogen module. DSC calibration for both temperature and enthalpy was performed using open-porous metallic standards (In, Pb, Zn). Aerogels were measured by weighing aliquots (ca. 1–5 mg) into 160 µL aluminum pans and submersing the samples in *o*-xylene. Measurements were carried out in ambient atmosphere using the following temperature program: (i) 0.7 °C min^−1^ from +25 to −70 °C, (ii) 0.7 °C min^−1^ from −70 to −30 °C ≤ T_x_ ≤ −20 °C, (iii) 0.7 °C min^−1^ from T_x_ to −70 °C and (iv) 0.7 °C min^−1^ from −70 to +25 °C. Three thermograms at least were acquired for each sample. Data processing was accomplished using STARe software.

Moisture sensitivity of the cellulosic aerogels, water uptake at different degrees of relative humidity (RH), and possible triggering of shrinkage of the cellulosic aerogels was studied at ambient temperature (20 ± 2 °C) in RH-controlled environment for a time period of 84 days after scCO_2_ drying. Desiccators used for these long-term studies were equipped with either phosphorus pentoxide (0% RH) or a saturated aqueous solution of CaCl_2_ (30% RH), NH_4_NO_3_ (65% RH), and K_2_SO_4_ (98% RH) to provide different levels of humidity after equilibration. The actual RH inside the desiccators was monitored by digital hygrometers (Voltcraft data logger DL−120TH) and was found to be largely constant (±5% RH). Throughout the entire experiment, weight (±1 mg) and volume (±0.1 mm^3^) of the aerogels were recorded periodically.

Uniaxial compression testing in longitudinal direction of the cylindrical aerogels (Ø = 10 mm, l = 20 mm) was performed on a Zwick/Roell Z010 equipment using a feed rate of 2.4 mm min^−1^. Only samples transferred immediately in argon atmosphere after scCO_2_ drying were used. Evaluation of the recorded stress strain curves was accomplished using testXpert software. Stiffness of the aerogels as represented by Young’s modulus *E* was determined from the slope of the regression line through the linear elastic region of each stress–strain curve; boundaries were adjusted individually for each curve. A 0.2% offset yield strength was recorded, i.e., the stress at the intersection of stress-strain curve and regression line through the linear elastic region shifted parallel by 0.2% strain as reported earlier [30].

Thermal analysis was conducted using STA 409 CD Skimmer equipment (Netzsch GmbH, Germany) allowing for simultaneous recording of thermogravimetric (TG) and differential scanning calorimetry (DSC) profiles. Aliquots of the samples (approx. 8–10 mg) were weighed into Al_2_O_3_ pans and combusted in helium atmosphere (120 mL min^−1^) using a constant heating rate of 10 °C min^−1^ over the entire temperature range of 30 to 900 °C.

## 5. Conclusions

Mixtures of tetra-*n*-butylammonium fluoride, DMSO, and small quantities of water can be used as an efficient, non-derivatizing solvent to prepare sufficiently low viscous dopes of cellulose phosphates (DS_P_ ca. 0.2) for further processing into lyogels and aerogels. At moderate dissolution conditions (60 °C, 4 h), this solvent system fully preserves the chemical integrity of the processed cellulosic materials. Care should be taken during work-up of the phosphorylated materials to ensure quantitative removal of non-reacted reagent and solvent components. Compared to aerogels from non-derivatized cellulose, cellulose phosphate aerogels suffer considerably less from shrinkage. This is presumably due to repulsive forces being effective throughout the entire solvent exchange procedure except for the last scCO_2_ drying step. With respect to the latter, it was interesting to observe that cellulose phosphate aerogels require shorter drying time to get rid of adhering ethanol. On the other hand, slightly increased surface polarity of the phosphorylated aerogels gives rise to a somewhat more pronounced sensitivity towards moisture sorption upon long-term storage. However, this drawback would be irrelevant if the materials would be used as lyogels instead. This would bear the advantage of circumventing that fraction of shrinkage that occurs during the scCO_2_ drying step. On the other hand, this would be at the expense of the better sterilization opportunities for aerogels. It can be summarized that phosphorylation targeting a low degree of substitution has no negative impact on key aerogel properties. This includes bulk density, pore size distribution, specific surface, or pore volume. Skin formation upon cellulose coagulation, on the contrary, is evidently less pronounced for cellulose phosphate aerogels. This would be beneficial for the design of dual-porous cell scaffolding materials, since their nanoporous struts separating interconnected large micron-size voids are supposed to maintain rapid transport of gases, nutrients and metabolic byproducts.

## Figures and Tables

**Table 1:** Phosphorous contents, DS_P_ values (degree of phosphorylation), and selected properties of 3% cellulose phosphate lyogels and aerogels compared to the non-derivatized starting materials. The range of variation represents the 95% confidence interval.

Sample	P Content [wt.%]	DS_P_	Shrinking (−) / Swelling (+)		
			Regeneration (EtOH, before Drying) [%]	After scCO_2_ Drying [%]	Apparent Density [mg cm^−3^]
hwPHK	-	-	−16.2 ± 4.9	−39.1 ± 5.5	58.1 ± 3.5
hwPHK-P	4.91	0.24	+4.0 ± 2.5	−28.3 ± 6.8	47.0 ± 5.8
CL	-	-	−17.8 ± 4.5	−45.7 ± 6.8	71.2 ± 5.0
CL-P	3.34	0.18	−2.4 ± 2.8	−31.4 ± 6.7	50.0 ± 4.0

**Table 2:** DS_P_ values of hwPHK-P (initial DS_P_ = 0.209) after extraction with DMSO or a solution of 16.6 wt.% TBAF in DMSO having a water content of 0.95 wt.% (labeled TBAF/DMSO) at room temperature and for different time periods. The range of variation indicates the 95% confidence interval (*n* = 3).

Extraction Medium	Extraction Time [h]	Final DS_P_	[%] of Initial DS_P_	
DMSO (control)	4	0.149	71	±0.0
TBAF/DMSO	2	0.094	45	±1.9
	4	0.093	44	±1.2
	8	0.096	46	±0.0
	8 (60 °C)	0.096	46	±0.0
	16	0.093	44	±0.5

**Table 3:** Young’s modulus (*E*), specific modulus (*E*_ρ_), yield strength (σ_y_), and yield stress (ε_y_) as obtained from uniaxial compression testing of the prepared aerogels.

Sample	*E* [MPa]	*E*_ρ_ [MPa cm^3^ g^−1^]	σ_y_ [MPa]	ε_y_ [%]
CL (TBAF/DMSO)	11.9	167	5.3	3.4
([EMIM][OAc]/DMSO) ^a^	1.121 ^a^	20 ^a^	2.1 ^a^	n.d. ^b^
NMMO⋅H_2_O ^a^	4.26 ^a^	68 ^a^	2.9 ^a^	n.d. ^b^
CL-P	10.8	216	3.6	5.1
hwPHK	5.9	102	5.7	7.6
hwPHK-P	3.2	68	1.9	3.4

^a^ data taken from [15], ^b^ not determined.

**Table 4:** Calculated porosity, results of nitrogen sorption experiments and pore characteristics derived from thermoporosimetry. Numbers in brackets indicate the 95% confidence interval (*n* = 3).

		Nitrogen Sorption Experiments			Thermoporosimetry	
Sample	Calculated Porosity [%]	Specific Surface [m^2^ g^−1^]	Sorbed Volume [cm^3^ g^−1^]	C Constant	V_p_ PSD [cm^3^ g^−1^]	R_p_ [nm] max PSD
CL	96.28	355 (43)	0.80 (0.20)	101 (1.0)	6.06	19.19
CL-P	96.99	311 (6.9)	0.55 (0.10)	48 (2.0)	9.27	21.23
hwPHK	95.46	366 (2.0)	0.7 (0)	104 (2.0)	7.32	19.74
hwPHK-P	96.80	270 (20)	0.5 (0)	45 (5.9)	10.60	24.49

**Table 5:** Volume and weight changes of studied aerogels in dependence on relative humidity and storage time (% of initial mass and volume right after scCO_2_ drying). Numbers in brackets indicate the 95% confidence interval (*n* = 4).

Sample	Conditioning [% RH]	2 d		84 d	
		Volume [%]	Weight [%]	Volume [%]	Weight [%]
CL	0	78.7 (1.3)	−4.2 (0.48)	68.0 (1.6)	−6.7 (0.25)
	30	72.0 (4.8)	−1.4 (0.06)	62.6 (5.2)	−1.5 (0.29)
	65	30.5 (3.0)	5.0 (1.0)	24.7 (2.9)	5.2 (0.63)
	98	10.0 (3.0)	23.6 (0.53)	10.4 (5.5)	25.8 (0.05)
CL-P	0	88.6 (1,4)	−1.7 (0.04)	78.9 (5.1)	−3.1 (0.13)
	65	27.4 (1.1)	6.6 (0.39)	19.7 (1.7)	5.8 (0.21)
hwPHK	0	85.6 (11)	−4.8 (0.37)	76.9 (8.4)	−4.9 (0.36)
	30	76.0 (6.5)	−0.3 (0.02)	68.7 (6.2)	−0.7 (0.13)
	65	36.3 (0.48)	5.5 (0.54)	27.2 (0.13)	5.4 (0.81)
	98	7.1 (0.72)	24.2 (0.01)	6.5 (0.08)	25.0 (0.07)
hwPHK-P	0	95.6 (5.2)	−0.6 (0.01)	87.6 (4.9)	−1.8 (0.01)
	65	30.9 (3.3)	6.1 (0.24)	21.4 (3.4)	4.8 (0.17)
